# Fluid–structure interaction simulation of the brain–skull interface for acute subdural haematoma prediction

**DOI:** 10.1007/s10237-018-1074-z

**Published:** 2018-08-27

**Authors:** Zhou Zhou, Xiaogai Li, Svein Kleiven

**Affiliations:** Neuronic Engineering, Royal Institute of Technology (KTH), Stockholm, Sweden

**Keywords:** Traumatic brain injury, Brain–skull interface, Fluid–structure interaction, Arbitrary Lagrangian–Eulerian, Finite element analysis

## Abstract

Traumatic brain injury is a leading cause of disability and mortality. Finite element-based head models are promising tools for enhanced head injury prediction, mitigation and prevention. The reliability of such models depends heavily on adequate representation of the brain–skull interaction. Nevertheless, the brain–skull interface has been largely simplified in previous three-dimensional head models without accounting for the fluid behaviour of the cerebrospinal fluid (CSF) and its mechanical interaction with the brain and skull. In this study, the brain–skull interface in a previously developed head model is modified as a fluid–structure interaction (FSI) approach, in which the CSF is treated on a moving mesh using an arbitrary Lagrangian–Eulerian multi-material formulation and the brain on a deformable mesh using a Lagrangian formulation. The modified model is validated against brain–skull relative displacement and intracranial pressure responses and subsequently imposed to an experimentally determined loading known to cause acute subdural haematoma (ASDH). Compared to the original model, the modified model achieves an improved validation performance in terms of brain–skull relative motion and is able to predict the occurrence of ASDH more accurately, indicating the superiority of the FSI approach for brain–skull interface modelling. The introduction of the FSI approach to represent the fluid behaviour of the CSF and its interaction with the brain and skull is crucial for more accurate head injury predictions.

## Introduction

Traumatic brain injury (TBI) has been regarded as a substantial global public health problem. In the USA, the annual number of TBI-related victims is estimated to be 1.7 million, resulting in 52,000 deaths, 275,000 hospitalizations and 1365 million dollars in medical cost (Faul et al. 2010). In Europe, epidemiologic studies reported an approximate TBI incidence rate of 262 per 10,000 (Peeters et al. 2015). Despite the substantial effort to reduce the occurrence and mitigate the consequences of TBI, a thorough understanding of the biomechanical injury mechanism appears elusive.

In recent decades, finite element (FE)-based head models have been growingly used to investigate the causation and effectuation of TBI. The biofidelity of such computational models requires a sufficient geometrically and mechanically detailed description of various anatomical structures, as well as precise representation of interactions between various intracranial components, such as the brain–skull interface (Goriely et al. 2015). Advances in medical imaging techniques have permitted brain and head models to incorporate a high degree of anatomical details. For example, several three-dimensional (3D) head models have successfully captured the morphological heterogeneities of the cerebral cortex in the form of the gyri and sulci (Chen and Ostoja-Starzewski 2010; Ghajari et al. 2017; Ho et al. 2009; Miller et al. 2016). In parallel, continuous experimental efforts have provided data for developing constitutive equations for numerical models. Kleiven (2007) modelled the brain material using a second-order Ogden-based hyperviscoelastic constitutive law with parameters fitted using data from brain tissue experiments in tension and compression by Franceschini et al. (2006) and in shear by Nicolle et al. (2005). Cloots et al. (2011, 2013) implemented an anisotropic fibre-reinforced material model to describe the anisotropic features of the brain tissue based on the shear experiments on brainstem along different directions with respect to axonal fibre orientation by Ning et al. (2006). To account for the nonlinear properties of the dura mater in the infant head, a Mooney–Rivlin hyperelastic model was employed by Li et al. (2017) with material parameters determined from tests of foetal dura mater reported in Bylski et al. (1986). However, current head models lack equivalent sophistication in brain–skull interaction representation.

Great disparity exists between models when it comes to brain–skull interaction modelling approaches, varying from representing the cerebrospinal fluid (CSF) as an incompressible material with a low shear modulus (Ho et al. 2009; Iwamoto and Nakahira 2015; Zhao et al. 2015; Zhou et al. 2016) to more complicated contact algorithms including tied contact (Mao et al. 2013; Zhou et al. 1995) and sliding-only contact (Kleiven and Hardy 2002). Of all these approaches, the CSF is consistently modelled by Lagrangian solid elements assuming no material transport (i.e. fluid flow). Since CSF is a biological fluid that flows within the subarachnoid space and ventricular system, using Lagrangian approach to model fluid or fluid-like material is often not appropriate, considering the large deformation that the material may experience. On the other hand, fluid element formulations, i.e. Eulerian formulation and arbitrary Lagrangian–Eulerian (ALE) formulation, allow mimicking the fluid behaviour of the CSF without causing severe element distortion. For the Eulerian formulation, the material flows through a mesh fixed in the space, while, for the ALE formulation, the material flows through a mesh moving according to a predefined directive.

To date, only a handful of two-dimensional (2D) FE models (Batterbee et al. 2011; Cheng et al. 2010; Fontenier et al. 2014; Gu et al. 2012) and simplified spherical 3D models (Klug et al. 2013) have implemented fluid elements for the CSF. The only 3D FE model with a realistic head geometry that modelled CSF as fluid element was reported by Willinger and Baumgartner (2003). Most of them have assumed a no-slip condition at the interface between the CSF and surrounding leptomeninges, given that merged interfacial nodes were commonly stated. However, this no-slip condition is applicable to the condition that the fluid mesh experiences small deformation. When the fluid mesh endures large deformation, e.g. the large deformation the CSF may experience at the brain–skull interface, this no-slip condition may cause excessive element distortion in the fluid mesh, which consequently results in low accuracy in the simulation result and small time step sizes for explicit time integration algorithm (Aquelet et al. 2006). An alternative to avoid or relieve severe fluid mesh distortion is to use a contact algorithm with allowing tangential sliding at the interface between the CSF and surrounding leptomeninges. Such a modelling strategy of employing fluid element for the CSF combined with sliding interface as coupling strategy has not been implemented in the existing 3D FE head models.

It is hypothesized that FSI simulation of the brain–skull interface leads to an improved model performance. Thus, the primary objective of this study is to implement an ALE multi-material formulation for the CSF and a contact algorithm to couple the CSF with the Lagrangian-represented brain in an existing FE head model. Model performances are evaluated by comparing brain–skull relative displacement–time histories and intracranial pressure–time histories with previously reported experimental data. A secondary objective is to assess the capability of this newly implemented FSI approach for acute subdural haematoma (ASDH) prediction by imposing the model to an experimentally determined loading known to cause ASDH. The relative motion between the brain cortex and the skull as well as bridging vein (BV) strain are used to assess the ASDH risk. Responses of the modified model are compared to those of the original model.

## Methods

### Finite element head model

**Fig. 1:**
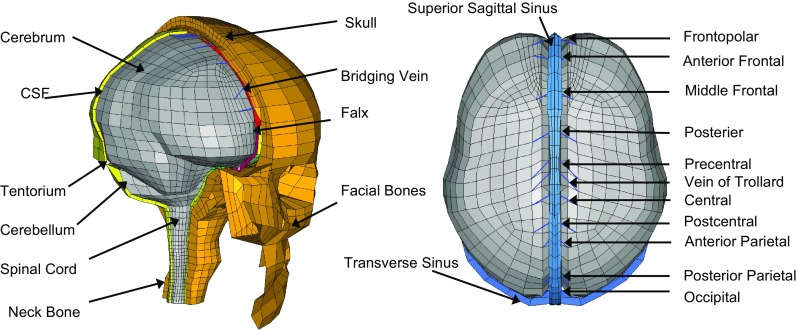
Finite element model of the human head (left) with the 11 pairs of bridging veins indicated (right)

The FE head model used in the current study was previously developed at the Kungliga Tekniska Högskolan (KTH) (Kleiven 2007). The head model includes the scalp, the skull, the brain, the meninges, the CSF, the ventricle, superior sagittal sinuses (SSS), a simplified neck with extension of the spinal cord and 11 pairs of parasagittal bridging veins (BVs) (Fig. 1). Material properties of each head component are available in Kleiven (2007) except for the membrane and CSF. The membrane material modelling is updated based on Ho et al. (2017). Briefly, instead of modelling the meninges with linear elastic materials, the pia mater is modelled with quasilinear viscoelastic material with material parameters derived from the experiment by Aimedieu and Grebe (2004); the falx and tentorium are modelled with simplified rubber/foam material based on the averaged stress–strain curves by Van Noort et al. (1981). A detailed description of CSF modelling is provided in the following section. The 11 pairs of parasagittal BVs are modelled as spring elements by connecting one node on the cerebral cortex to the other on the SSS. The length and angle of each modelled BV are listed in Table 1.

**Table 1:** Summary of BV length and resultant angles from the mid-sagittal line to the BV elements in the counterclockwise direction

Bridging veins	Length (mm)	Angle (degree)
Frontopolar	16.5	87
Anterior frontal	15.8	103
Middle frontal	9.7	69
Posterior frontal	13.7	64
Vein of Trolard	18.4	47
Precentral	18.2	46
Central	14.9	61
Post-central	15.0	59
Anterior parietal	8.8	44
Posterior parietal	19.9	66
Occipital	17.8	74

In the original model (referred to as sliding-model), a sliding-only contact, which permits sliding in the tangential direction, was introduced between the CSF and the brain to mimic the brain–skull interface (Kleiven 2007). The CSF was modelled by Lagrangian solid elements as an elastic fluid constitutive model in LS-DYNA (LSTC, Livermore, CA) (Hallquist 2007). The pressure (*P*) in the CSF elements is expressed as:

1 $$ \dot{P} = - K \dot{\varepsilon }_{ii} , $$

where $\dot{P}$ is the pressure rate, *K* is the bulk modulus, and $\dot{\varepsilon }_{ii}$ is the dilatational strain rate.

As previously reported in Kleiven (2007), the *K* is set as 2.19 GPa. Although the shear modulus of the CSF is set to zero, the deviatoric stress (*σ*_*ij*_^*v*^) is calculated as:

2 $$ \sigma_{ij}^{v} = VC \cdot \Delta L \cdot a \cdot \rho \dot{\varepsilon }_{ij}^{\prime } , $$

where Δ*L* is the characteristic element length, *a* is the fluid bulk sound speed by $a = \sqrt {\frac{K}{\rho }}$, *ρ* is the fluid density, $\dot{\varepsilon }_{ij}^{\prime }$ is the deviatoric strain rate, and *VC* is a tensor viscosity coefficient.

It is worth clarifying that the *VC* is a numerical damping coefficient to suppress spurious oscillations and is irrelevant to the fluid dynamic viscosity. According to the recommended value range (0.1–0.5) by Hallquist (2007), *VC* is defined as 0.3 in the sliding-model.

### Implementation of FSI for the brain–skull interface

**Fig. 2:**
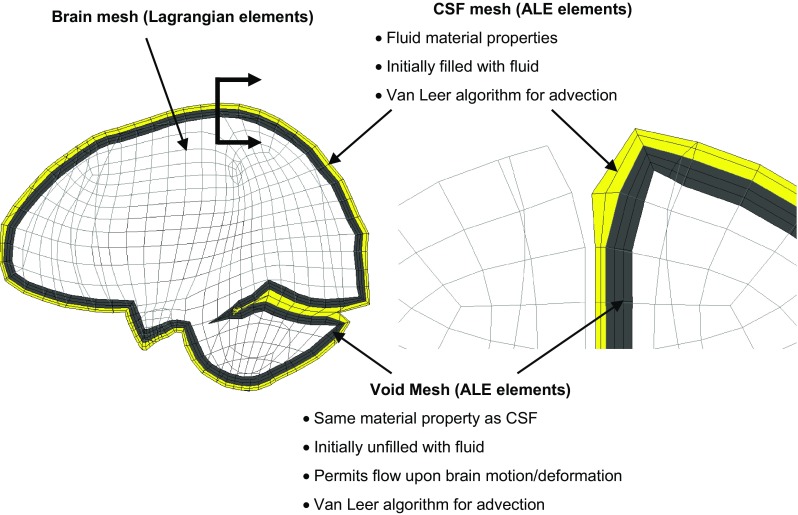
Mesh configuration and material properties for the ALE elements. Sagittal section of the head model (left) and a magnified coronal section masking half of the ALE elements surrounding the brain for a better illustration (right)

To model the fluid properties of the CSF and potential CSF flow, CSF elements in the subarachnoid space are modified using ALE multi-material formulation (Hallquist 2007). A mesh sensitivity study shows that two-layer elements for the CSF in the thickness with an equivalent element size as that of brain elements in the CSF–brain interface is adequate to achieve converged brain responses (Appendix A). Thus, all results reported in this study are obtained from the model with the aforementioned CSF mesh. Following the requirement of FSI implementation, any locations to which CSF may potentially flow during the simulation are required to be meshed. It is expected that the CSF may be transported to the space initially occupied by the brain cortex due to brain deformation and brain–skull relative motion. Thus, supplemental mesh, referred to as void mesh, is generated in these regions in HYPERMESH (13.0, Altair, Troy, MI, USA) (Fig. 2). The void mesh is assigned the same material property and element formulation as the CSF elements with additionally specifying a void option to ensure that no fluid material is filled in the void mesh at the initial configuration (Hallquist 2007). The void mesh is similarly introduced in other FSI studies (Luraghi et al. 2017; Stitzel et al. 2002; Zhang and Suzuki 2006). Motion of the ALE elements follows the mass-weighted averaged velocity in the ALE mesh (Hallquist 2007).

The ALE formulation advances the solution in time using operator splitting into two phases. First, a Lagrangian phase is performed wherein the deformation of the FE mesh follows exactly the deformation of the material secondary to the internal and external forces, i.e. involving mesh deformation. This Lagrangian step is related to the equation of state (EOS) together with constitutive equations. The EOS governing the dilatational behaviours of the CSF is modelled using the nonlinear Mie–Gruneisen EOS (Hallquist 2007), same as the modelling choice in previous FSI studies (Batterbee et al. 2011; Fontenier et al. 2014; Gu et al. 2012). The pressure in the CSF is expressed as follows:

3 $$ P = \frac{{\rho_{0} C^{2} \mu \left[ {1 + \left( {1 - \frac{{\gamma_{0} }}{2}} \right)\mu - \frac{a}{2}\mu^{2} } \right]}}{{\left[ {1 - \left( {S_{1} - 1} \right)\mu - S_{2} \frac{{\mu^{2} }}{\mu + 1} - S_{3 } \frac{{\mu^{3} }}{{\left( {\mu + 1} \right)^{2} }}} \right]}}, $$

where *C* is the intercept of *v*_*s*_–*v*_*p*_ curves with *v*_*s*_ being the velocity of a shockwave travelling through the intermediary material and *v*_*p*_ being the velocity of the shocked material; *S*_1_, *S*_2_ and *S*_3_ are the coefficients of the slope of the *v*_*s*_–*v*_*p*_ curves; *γ*_0_ is the Gruneisen gamma; and *a* is the first-order volume correction to *γ*_0_; *μ* is defined as:

4 $$ \mu = \frac{{V_{0} }}{V} - 1 = \frac{\rho }{{\rho_{0} }} - 1, $$

where *V*_0_ is the initial volume, *V* is the instantaneous volume, *ρ* is the instantaneous density, and *ρ*_0_ is the initial density.

Since CSF is a water-like fluid, the standard EOS constants for water are employed with *C *= 1482.9 m/s, *S*_1_ = 2.1057, *S*_2_ = − 0.1744, *S*_3_ = 0.010,085, *a *= 0, and *γ*_0_ = 1.2 (Dobratz 1981).

The deviatoric response of the CSF is determined by its constitutive modelling, expressed by

5 $$ \sigma_{ij}^{v} = \gamma \dot{\varepsilon }_{ij}^{\prime } , $$

where *γ* is the dynamic viscosity.

The CSF constitutive behaviour is modelled by a fluid material model (Hallquist 2007) with a dynamic viscosity of 0.001 Pa.s (Bloomfield et al. 1998). The cut-off pressure is a parameter that must be defined to limit the magnitude of tensile pressure at the contrecoup site. As detailed in “Appendix B,” to avoid the unphysical brain–skull decoupling at the contrecoup site, the value is set as − 22 MPa, similar to the cavitation strength of degassed water at 36 °C (Herbert et al. 2006).

In the second phase, the element state variables are transported back to the reference domain, including mass transport, momentum and energy convection across the element boundaries. This advection effect may result in mass fluxes flowing through the mesh. The details regarding the advection algorithm can be found in Benson (1989) where the first-order donor cell method and the second-order van Leer method were presented. Here, the second-order van Leer scheme is selected (Fig. 2), excelling in advection accuracy (Van Leer 1997).

For the interface between the dura and skull, a tied contact is imposed given that the dura is tightly adhered to the skull inner surface. At the interface between the CSF (ALE solid elements) and pia mater (Lagrangian shell elements), a penalty-based contact is defined (Hallquist 2007). The chosen contact allows sliding in the tangential direction and delivering tension and compression load in the radial direction. Following the approach of Batterbee et al. (2011), the CSF outer surface is merged with the dura mater since the CSF can only flow within the space encompassed by dura mater in the absence of skull fracture injury.

### Model validation

The localized motion response of the modified head model (referred to as FSI-model) is validated against available post-mortem human subject (PMHS) experiments by Hardy et al. (2007), where the local displacement identified by the implanted neutral density targets (NDTs) was measured using a high-speed X-ray under the impact scenarios. In the present work, three representative cases are selected, including C288-T3 (sagittal impact), C380-T4 (coronal impact) and C380-T5 (horizontal impact). To reproduce the geometry of the specimen at utmost, the models are scaled independently in directions of both the depth and breadth to match the reported cadaveric head sizes. The recorded head kinematic curves are imposed to the nodes at the centre of gravity of the corresponding PMHS and are rigidly attached to the skull. Relative displacements along all three anatomical coordinate directions for a total of 42 NDTs are obtained from the model and compared with the measurements.

**Fig. 3:**
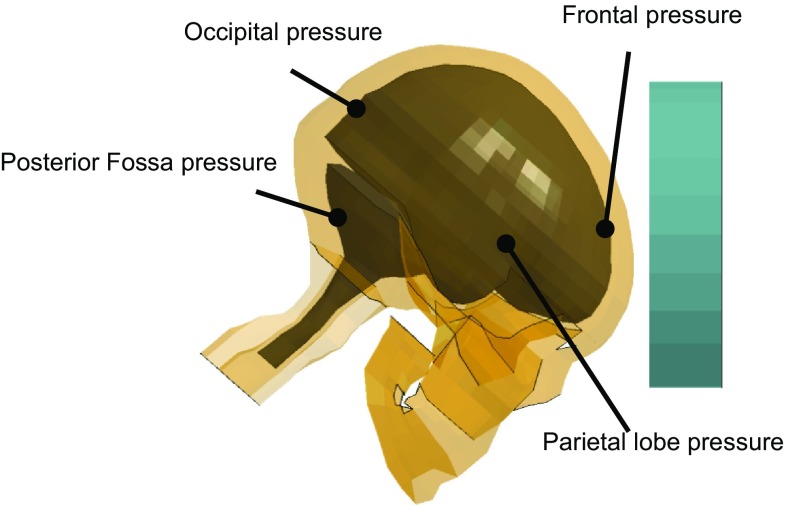
Pressure-measuring sites in the simulation of experiment No. 37 conducted by Nahum et al. (1977)

Intracranial pressure response of the FSI-model is compared with recordings from experiment No. 37 conducted by Nahum et al. (1977), where impacts to the forehead by a padded impactor were performed and pressure secondary to the delivered impact was measured at four sites: (1) frontal lobe adjacent to the impact contact area; (2) inferior to the lambdoidal suture in the occipital bone; (3) immediately posterior and superior to the coronal and squamosal suture in the parietal area; (4) posterior fossa in the occipital area. Following the experiment setting, the model is scaled to match the dimension of the corresponding cadaveric head and rotated 45° forward. Adopting the strategy of Kleiven (2006) regarding blow delivery, the mass and geometry of cylindrical impactor and the interposed padding materials at the impacting end are developed. Due to the unavailability of material details of the padding, the padding material parameters are adjusted to ensure the impact force and acceleration characteristics of the experiment are largely reproduced. As shown in Fig. 3, pressure–time history curves of the cortical brain elements at the four aforementioned locations are compared with the measurements. Such a strategy for intracranial pressure validation is similarly adopted in Kleiven (2006) for the sliding-model, as well as in other previously reported studies (Claessens et al. 1997; Horgan and Gilchrist 2003; Zhao et al. 2015).

The FSI-model validation performance is compared with that of the sliding-model, which has been previously reported in Giordano and Kleiven (2016) for the brain–skull relative motion validation and in Kleiven (2006) for the intracranial pressure validation. The performances of both models are evaluated using a matrix called CORA (CORrelation and Analysis). The technical details are available in Gehre et al. (2009). Briefly, the CORA assesses the level of correlation between a pair of time history curves by a combination of corridor rating (C1) and cross-correlation rating in terms of shape (V), size (G) and phase (P). An overall biofidelity rating (B) is calculated by averaging the scores of the four evaluating terms and categorized into five classifications (Table 2).

**Table 2:** Biofidelity classification and corresponding ranges of overall biofidelity rating score (B)

Biofidelity classification	B range
Excellent	8.6–10
Good	6.5–8.6
Fair	4.5–8.5
Margin	2.6–4.5
Unacceptable	0.0–2.6

### ASDH predictability assessment

**Fig. 4:**
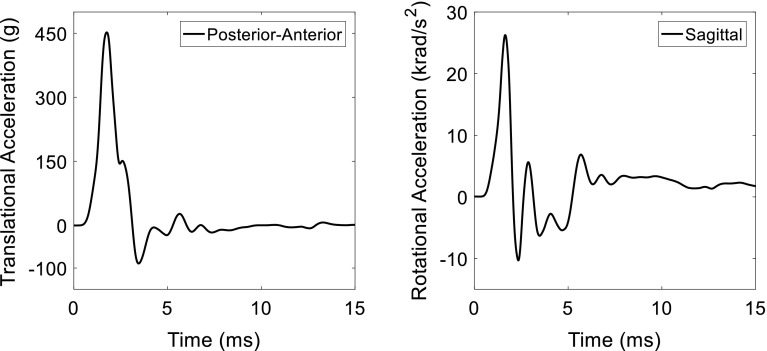
Resultant translational acceleration (left) and rotational acceleration loading curves applied to the head model

Previous numerical studies suggest that brain–skull interface modelling approaches have direct impacts on the prediction of meningeal-related injury, such as ASDH resulting from BV rupture (Jin et al. 2014; Kleiven 2003). Thus, it is important to evaluate the ASDH predictability of this newly implemented FSI approach. An important experimental determination of human tolerance level for BV-induced ASDH so far is conducted by Depreitere et al. (2006) by delivering occipital impacts in sagittal plane to the cadaver. Detailed experimental loading curves have been reported recently by Cui et al. (2017). One representative case (21-2) with detected BV rupture in the cadaver is selected. The recorded head kinematic curves (Fig. 4) are imposed to the rigidly modelled skull in the FSI-model. The peak translational acceleration is 450 g, and the peak angular acceleration is 26.2 krad/s^2^. The same loading is exerted to the sliding-model, and the corresponding responses are compared to those of the FSI-model.

## Results

For the simulation, a selectively reduced (S/R) integration scheme and hourglass control are used for the brain and CSF in all simulations. The hourglass energies are always controlled to be lower than 10% of the total energy in both models. In particular for the CSF, the ratio of the hourglass energy to the total energy is under 10% as well. For a simulation of 40 ms, it typically requires 9 h for the FSI-model, and 5 h for the sliding-model using a local Linux cluster with a single processor. LS-DYNA version ls971_s_R5_1_1_amd64_suse10 is used.

### Validation results

The CORA rating scores for brain–skull relative displacement estimation of the FSI-model and the sliding-model are presented in Table 3. B values of the FSI-model for the three representative experiments are 5.30 for the sagittal impact (C288T3), 5.60 for the coronal impact (C380T4) and 6.27 for horizontal impact (C380T5), respectively. The average values of all the three cases for each evaluating term are 7.17 for V, 4.25 for G, 7.57 for P, and 3.83 for C1, ultimately contributing to an overall B value of 5.72, which is relatively higher than that of the sliding-model (4.91). Brain–skull relative motion responses between the models and the experimental recordings are provided in “Appendix C”.

**Table 3:** Summary of biofidelity ratings derived from CORA regarding model validation performance of brain–skull relative displacements

Case	V		G		P		C1		B	
	FSI-model	Sliding-model	FSI-model	Sliding-model	FSI-model	Sliding-model	FSI-model	Sliding-model	FSI-model	Sliding-model
C288T3	6.43	5.97	3.75	1.95	7.60	7.22	3.27	2.48	5.30	4.40
C380T4	7.24	6.02	4.99	4.10	6.51	5.63	3.65	2.82	5.60	4.65
C380T5	7.85	7.67	4.01	3.97	9.50	7.07	4.59	4.04	6.27	5.69
Average	7.17	6.55	4.25	3.34	7.87	6.64	3.83	3.31	5.72	4.91

**Fig. 5:**
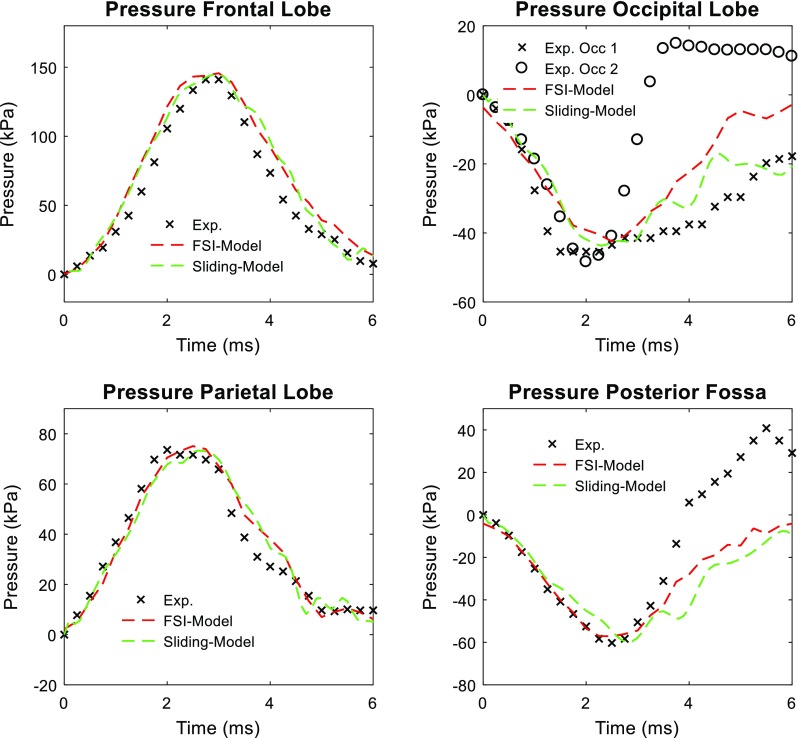
Comparison between experimental data and simulated intracranial pressure for experiment No. 37 conducted by Nahum et al. (1977)

The CORA rating scores for intracranial pressure of the FSI-model and the sliding-model at frontal, parietal and fossa sites are compared in Table 4. Of all the three sites, both the FSI-model and the sliding-model have comparable B values larger than 8.6, denoting excellent biofidelities in pressure reponse. For the occipital site (Fig. 5), no attempt is made to calculate the CORA scores, given the obvious differences between the measured data at two occipital sites (Exp. Occ1 and Exp. Occ2 in Fig. 5) and the unavailability of accurate measuring locations. Nevertheless, the model-estimated pressure falls within the range defined by the experimental data, indicating the plausibility of pressure response at the occipital lobe.

**Table 4:** Summary of biofidelity ratings derived from CORA regarding model validation performance of intracranial pressure

Site	V		G		P		C1		B	
	FSI-model	Sliding-model	FSI-model	Sliding-model	FSI-model	Sliding-model	FSI-model	Sliding-model	FSI-model	Sliding-model
Frontal	9.92	9.99	7.75	9.52	9.99	9.99	9.99	9.99	9.41	9.87
Parietal	9.92	9.99	9.44	8.90	9.99	9.99	9.99	9.99	9.83	9.71
Fossa	8.96	9.99	9.32	8.64	9.99	9.99	7.69	7.67	9.06	9.07
Average	9.60	9.99	8.88	9.02	9.99	9.99	9.32	9.21	9.43	9.55

### ASDH predictability assessment results

**Fig. 6:**
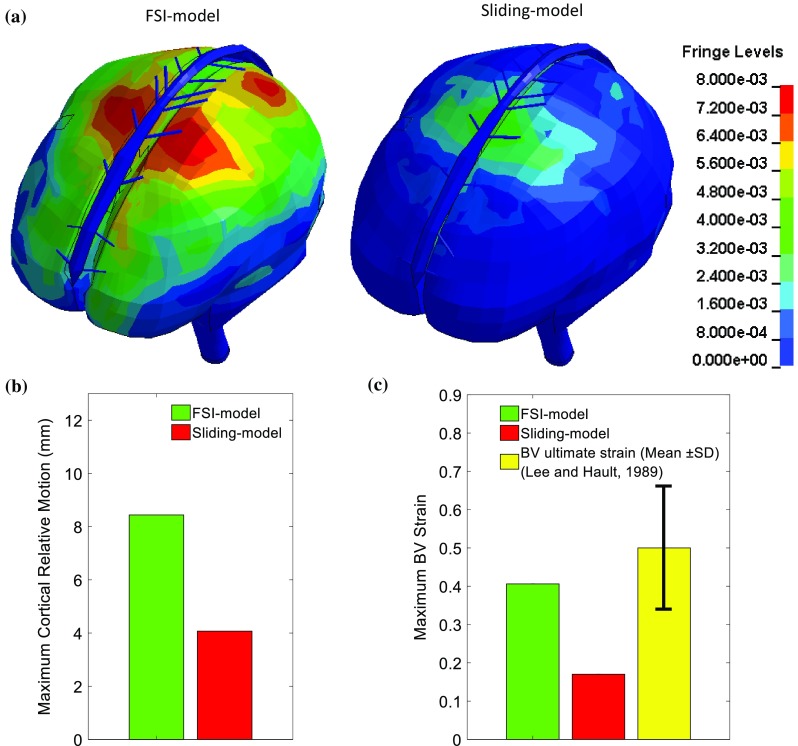
Cortical relative motions and BV strains predicted from the FSI-model and the sliding-model. **a** Cortical relative motion captured at the maximum value occurring time using a colour scale in unit of metre. **b** Maximum value of the cortical relative motion. **c** Maximum BV strain plotted together with the BV ultimate strain from Lee and Haut (1989). SD represents standard deviation

Following the strategy of Kleiven (2003), cortical relative motion (relative motion between the cerebral surface and the skull) and BV strain are extracted to assess the ASDH risk. In both models, the maximum cortical relative motions coincidently locate at the posterior frontal and precentral regions (Fig. 6a). While considerably larger cortical relative motion up to 8.44 mm is predicted by the FSI-model, a much lower cortical relative motion is predicted by the sliding-model with a peak value of 4.07 mm (Fig. 6b). The maximum BV strains are found at anterior parietal regions in both models. For the FSI-model, the maximum BV strain is 0.41, falling within BV ultimate strain range (0.5 ± 0.16) reported by Lee and Haut (1989). This finding indicates a possible ASDH occurrence due to the BV failure, in accordance with the experimental observation. For the sliding-model, the estimated BV strain peak (0.17) is even less than the lowest BV rupture strain (0.36) from Lee and Haut (1989), implying the predicted BV strain is insufficient to cause BV failure.

**Fig. 7:**
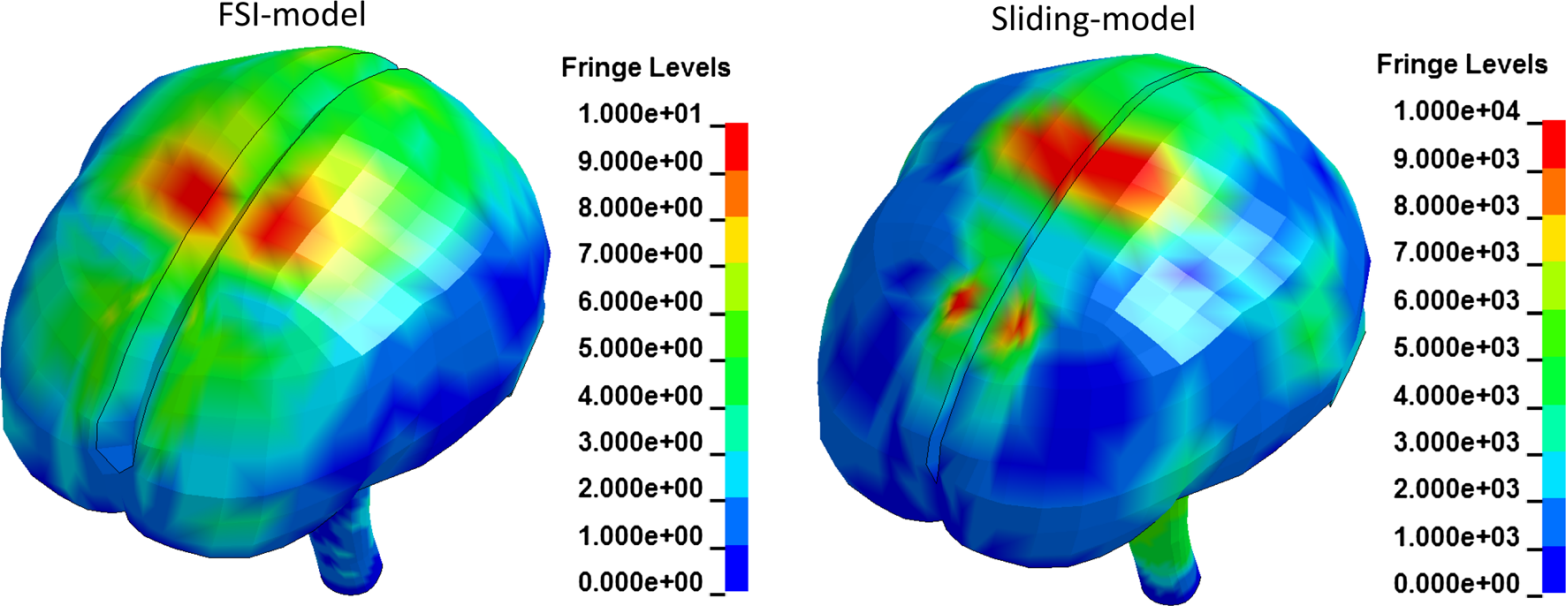
Contours of shear stress in the CSF predicted by the FSI-model (left) and the sliding-model (right) at the maximum cortical relative motion occurring time using a colour scale in unit of Pa

Figure 7 shows the shear stress in the CSF at the maximum cortical relative motion occurring time. It can be seen that the shear stress in the CSF predicted by the sliding-model is about three orders larger in magnitude than that by the FSI-model.

## Discussion

The present study details the implementation of a FSI approach for simulating the brain–skull interaction in a 3D head model by coupling the Lagrangian-represented brain elements with the ALE-represented CSF elements. The modified head model is validated in terms of brain–skull relative motion and intracranial pressure and subsequently subjected to an experimentally determined injurious loading for ASDH predictability assessment. Compared to the original KTH head model, the modified KTH head model exhibits an improved validation performance quantified by CORA and an enhanced correlation between model-predicted ASDH occurrence and experimental observation. These results indicate the superiority of the FSI approach for the brain–skull interface representation.

In head impact computational simulations, classical Lagrangian FE methods are the dominant numerical schemes. When it comes to the large deformation regime under severe impact, a pure Lagrangian approach may not be sufficient. Especially for the CSF, using Lagrangian-based representation may cause excessive distortion and unnatural resistance, ultimately leading to numerical instability and biased computational prediction (Darvish and Crandall 2002). As an alternative approach, ALE formulation can resolve the mesh distortions. This formulation allows the FE mesh to be controlled following the moving boundaries under specified mode and preserve element shape, as opposed to the Lagrangian formulation where the mesh follows exactly material deformation. Thus, in the current study, the ALE multi-material formulation is used to model CSF with its material behaviour determined by an EOS and a constitutive equation. A penalty-based approach is used to couple the ALE-modelled CSF response to the Lagrangian-modelled brain response. The basic idea of the penalty formulation is that the pial surface acts as a moving boundary to the CSF, iteratively calculating the relative position between the coupled Lagrangian structure and the ALE fluid within each time step. Once a penetration is detected, a coupling force proportional to the penetrating distance is applied to the fluid and structure. Consequently, the coupling algorithm allows CSF to flow only around and along brain, but not through the brain (Souli et al. 2000).

Cut-off pressure is parameter needed in the modelling of CSF as fluid elements. The proposed cavitation pressure in the literature, in which either water- or gel-like material was used as a substitute of the CSF, varies several orders in magnitude. It has been reported that cavitation of tap water was close to 1 atmosphere (− 100 kPa) (Herbert et al. 2006), supported by the claim that most fluids depend largely on external atmospheric pressure to hold them together due to the absence of cohesive strength. Once the negative pressure approaches the ambient pressure (near-zero absolute pressure), cavities will form due to the vapour pressure of the fluid (Al-Bsharat et al. 1999). Recently, a comparable cavitation threshold (− 140 kPa) under the blast scenario has also been determined by fluid-filled skull surrogation (Salzar et al. 2017). Although these experiments with fluid-filled containers have indeed lent credence to uncover the cavitation occurring mechanism, the pressure level at which CSF cavitation occurs may not be the same, considering the constituent difference and the additional trabeculae structure in the subarachnoid space. Salzar et al. (2017) explicitly stated that the cavitation pressure for a living specimen might be significantly larger, in magnitude, than − 140 kPa, given more nucleation sites existed in the CSF. This is also stated by Lubock and Goldsmith (1980), who argued that the removal of nucleation sites could substantially raise the cavitation strength to values up to 41 MPa. Moreover, cavitation occurrence in CSF itself or brain–skull interface has never been experimentally or clinically observed, neither in PHMS nor in living human. It remains to be verified that CSF cavitation indeed occurs in the intracranial cavity secondary to exterior impact (Panzer et al. 2012). Thus, the cut-off pressure value in the CSF should be assigned with care. Here, to avoid the potential numerical instability associated with the unphysical brain–skull decoupling at the contrecoup site (Felippa and DeRuntz 1984), a cut-off pressure of the degassed water is selected in the current study.

The modified model is validated regarding brain–skull relative motion and intracranial pressure and compared with the original sliding-based KTH head model. For the brain–skull relative motion, the FSI-model has an overall enhanced validating performance. The B values of the FSI-model increase by 20.4% for the sagittal impact, 20.4% for the coronal impact, and 10.1% for the horizontal impact. On average, the CORA rating scores for the FSI-model increase 9% for V, 27% for G, 18% for P, as well as 14.6% for C1, resulting in an overall increase in B of 16%. For the intracranial pressure, both models have excellent biofidelity. The enhanced biofidelity of the FSI-model indicates the importance of accounting for the CSF fluid behaviour and its mechanical interaction with the brain in head models. It is worth clarifying that the formula for calculating the CORA value for the sliding-model has been modified to account for both the corridor rating and cross-correlation rating, instead of solely considering the cross-correlation rating as in Giordano and Kleiven (2016).

When proposing a new modelling technique for the brain–skull interface, it is important to assess its applicability in terms of head injury prediction, especially for ASDH. For example, it has been questioned by Kleiven (2003) of using a model with tied contact for the brain–skull interface to predict ASDH given that the tied interface rules out the possibility of any large relative motion between the skull and brain. When approximating the brain–skull interaction as a layer of CSF with sharing nodes with neighbouring structures, it is hardly possible to predict injurious BV strains solely relying on the deformation of the thin CSF layer while achieving numerical reliability. This suspicion is supported by Al-Bsharat et al. (1999) in which it is stated that approximating the brain–skull interaction as a layer of low shear material without any sliding between head components was not able to predict brain–skull relative motion over 1 mm.

The ASDH predictability of the FSI-model is assessed by imposing an ASDH injurious loading to the models. The cortical relative motion peak estimated by FSI-model is larger than that by the sliding-model. This could be explained by the fact that different strategies are employed to compute deviatoric stresses in the CSF. Compare to the sliding-model, the FSI-model predicts significantly smaller stress magnitude in the CSF, which realistically reflects the low shear resistance properties of the fluid. Because of this, less dose of contact force is needed in the FSI-model to reach the local equilibrium at the contact interface, which ultimately allows the less constrained brain to slide more easily in the FSI-model. The maximum BV strain predicted by the FSI-model falls within the reported BV ultimate strain range in the literature, suggesting that the FSI approach can be used for ASDH-related studies. Although the sliding-only contact applies penalty-based algorithm to calculate the brain–skull interacting force in common with the FSI approach, the BV strain peak predicted by the sliding-model fails to reach the injurious level. This further indicates the importance of accounting for the fluid properties of CSF. Lack of correlation between the maximum cortical relative motion site and BV rupture site is found in both models, as also observed in Kleiven (2003). This inconsistency can be explained by the anatomical differences in length and orientation between the BVs.

**Fig. 8:**
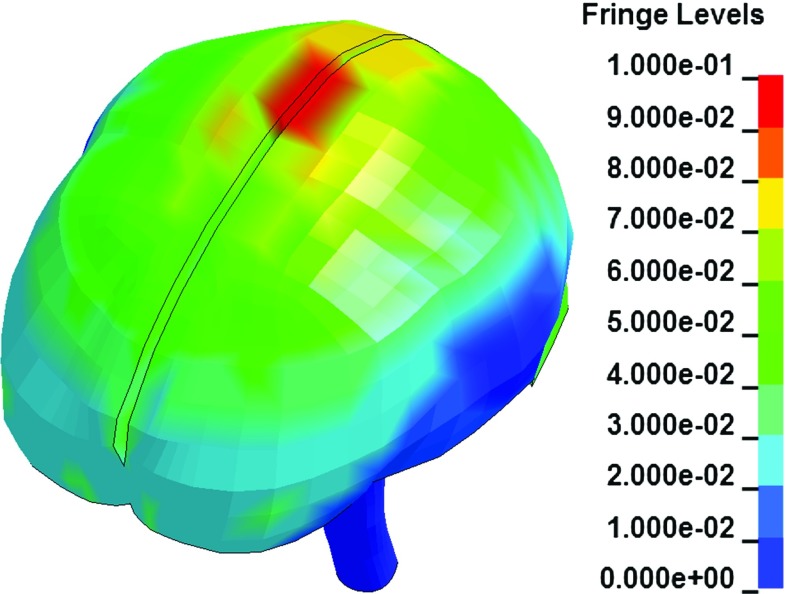
Contour of max shear Green–Lagrange strain in the CSF predicted by the sliding-model at the maximum cortical relative motion occurring time

To ensure the reliability of the simulation results by the sliding-model in which the CSF is modelled by Lagrangian elements, the CSF element distortion is checked. Following the approach in Larson et al. (2002), shear strain is selected as the indicator of element distortion severity. Figure 8 plots the max shear Green–Lagrange strain in the CSF at the maximum cortical relative motion occurring time with a peak of 0.17. Since the CSF strain is rarely reported in the literature, this peak is alternatively compared to the shear strain in the brain. In light of the findings that the brain is commonly reported to experience shear strain over 0.3 in the head impact simulation (Fahlstedt 2015; Kimpara and Iwamoto 2012; Mao and Yang 2011; Zhang et al. 2002), the CSF element distortion in the sliding-model is acceptable.

Compared to the study by Willinger and Baumgartner (2003) in which the CSF was modelled by ALE elements with a no-slip condition, severe element distortion is avoided in the current study owing to the permission of tangential sliding in the CSF–brain interface. Moreover, only the pressure response of the model by Willinger and Baumgartner (2003) was validated. However, a model validated against brain pressure response alone cannot be used to predict head injury associated with brain–skull relative motion, such as ASDH (Bradshaw and Morfey 2001; Kleiven and Hardy 2002; Zhao et al. 2015).

## Limitation and future work

The study has some limitations. First, the tethering effects of the sheet-like trabeculae structure within the subarachnoid space are not considered. There is only one quantitative study so far by Scott et al. (2016) that investigated the influence of arachnoid structure on the extra-axial haemorrhage in the immature piglet by incorporating the mechanical properties of the pia–arachnoid complex measured from bovine (Jin et al. 2006, 2007, 2011) and morphological information of the leptomeninges characterized from porcine (Scott and Coats 2015). Considering the inter-species variations, any extrapolation of animal study results to the human head needs further verification. Thus, the mechanical role of the arachnoid trabeculae for the brain–skull interaction in the human head remains to be justified. Secondly, due to the limited topological information, only 11 pairs of largest parasagittal BVs are incorporated in the models. However, the smaller BVs might be additional bleeding sources responsible for the development of ASDH. It is recommended that a more realistic BV topological representation be incorporated when detailed BV anatomical information is available.

## Conclusion

This paper presents the essential effort of implementing a FSI approach to model the brain–skull interaction in the KTH head model. The modified model is validated against brain–skull relative displacement–time histories and intracranial pressure–time histories and evaluated in terms of predicting ASDH by subjecting the model to an experimentally determined injurious loading. Compared to the original KTH head model, the enhanced model validating the performance and the improved ASDH predictability of the modified KTH head model suggests it is more appropriate to adopt the FSI approach representing the brain–skull interaction.

## Figures and Tables

**Fig. 9:**
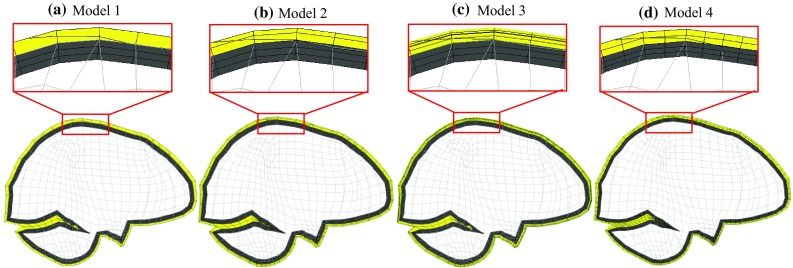
Sagittal section of the brain model (low row) together with a magnification (top row) showing the initial CSF-filled volume in yellow with varying element layers and aspect ratio (ALE elements), initially unfilled volume in dark grey (ALE elements) and brain in light grey (Lagrangian elements)

**Fig. 10:**
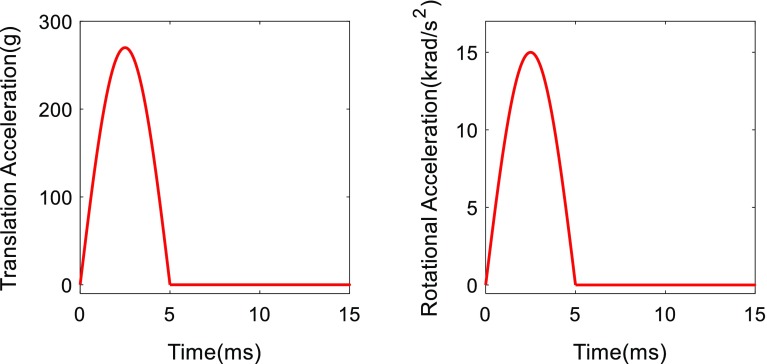
Acceleration loading curves applied to the head models

**Fig. 11:**
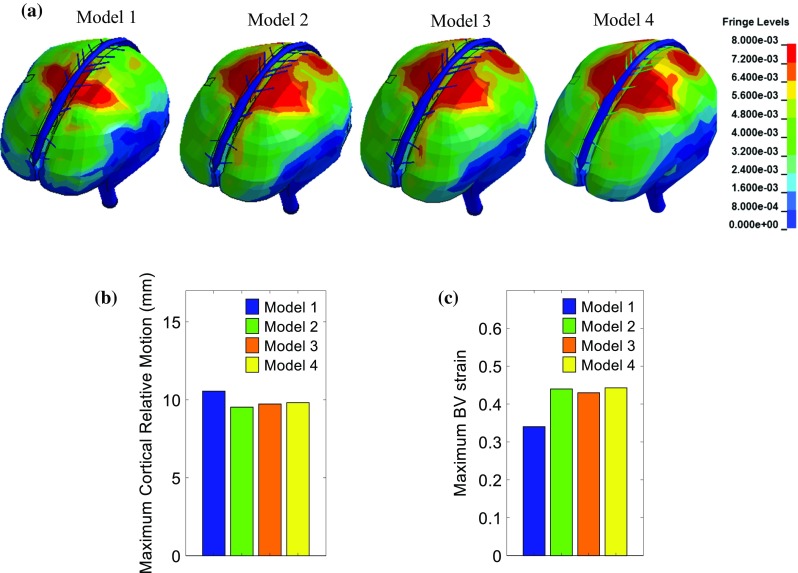
Result of the cortical relative motions and BV strains of three models. **a** Cortical relative motion distribution at the maximum value occurring time using a colour scale in unit of metre. The bar chart **b** shows the maximum value of the cortical relative motion. The bar chart **c** shows the maximum BV strain

**Fig. 12:**
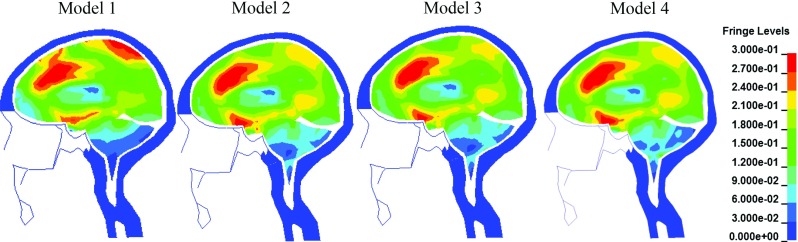
Sagittal cross section of the first principal Green–Lagrange strain for the four models

**Fig. 13:**
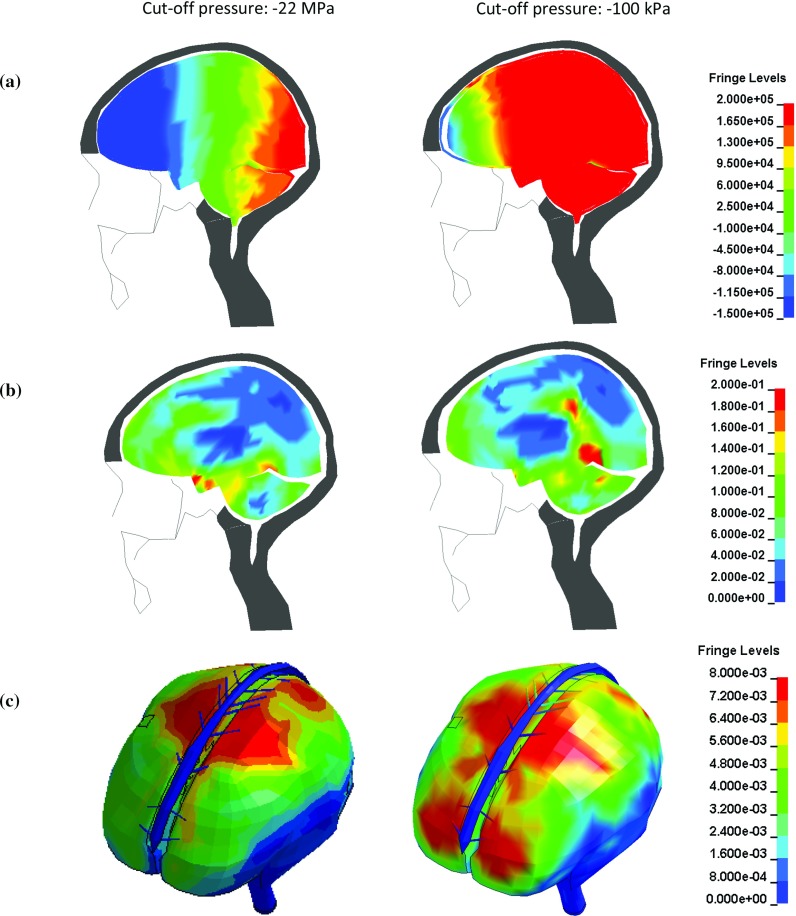
Results of intracranial responses for models with cut-off pressure of the CSF as − 22 MPa and − 100 kPa. **a** Contour of intracranial pressure at 2.5 ms using a colour scale with a unit of Pa; **b** Sagittal cross section of first principal Green–Lagrange strain at 2.5 ms using a colour scale. **c** Cortical relative motion distribution using a colour scale with a unit of mm at its maximum value occurring time. Subfigures **a** and **b** illustrate the skull coloured in dark grey as a reference frame. The CSF is plotted in subfigure (**a**) to better visualize the brain–skull decoupling

**Fig. 14:**
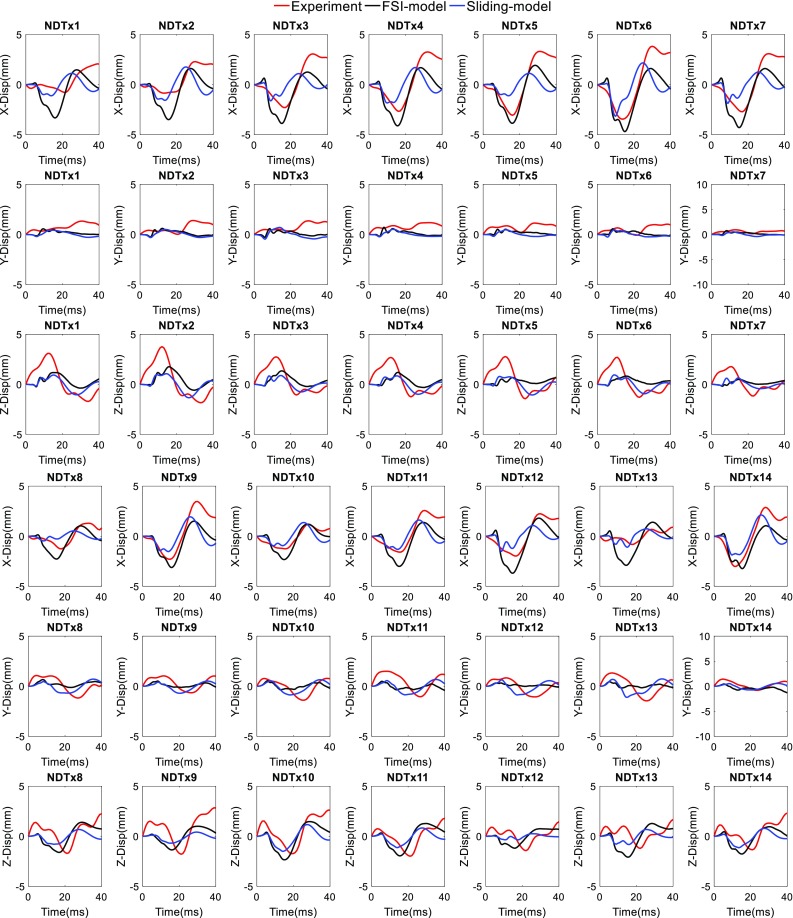
Comparison between experimental data and simulated brain–skull relative displacement for the experiment C288-T3

**Fig. 15:**
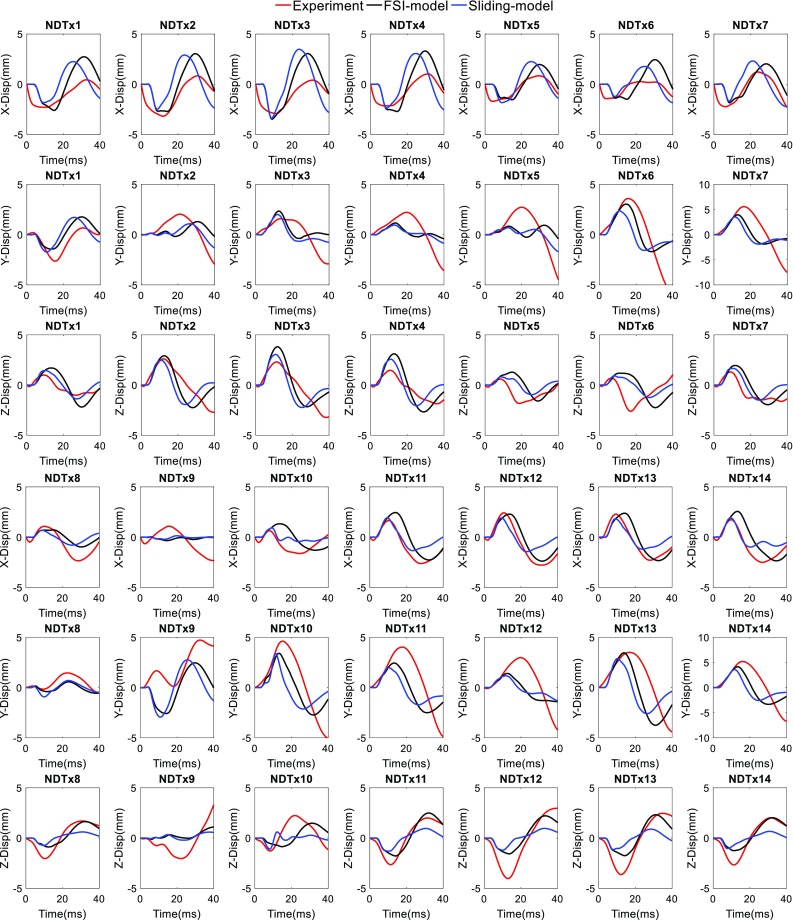
Comparison between experimental data and simulated brain–skull relative displacement for the experiment C380-T4

**Fig. 16:**
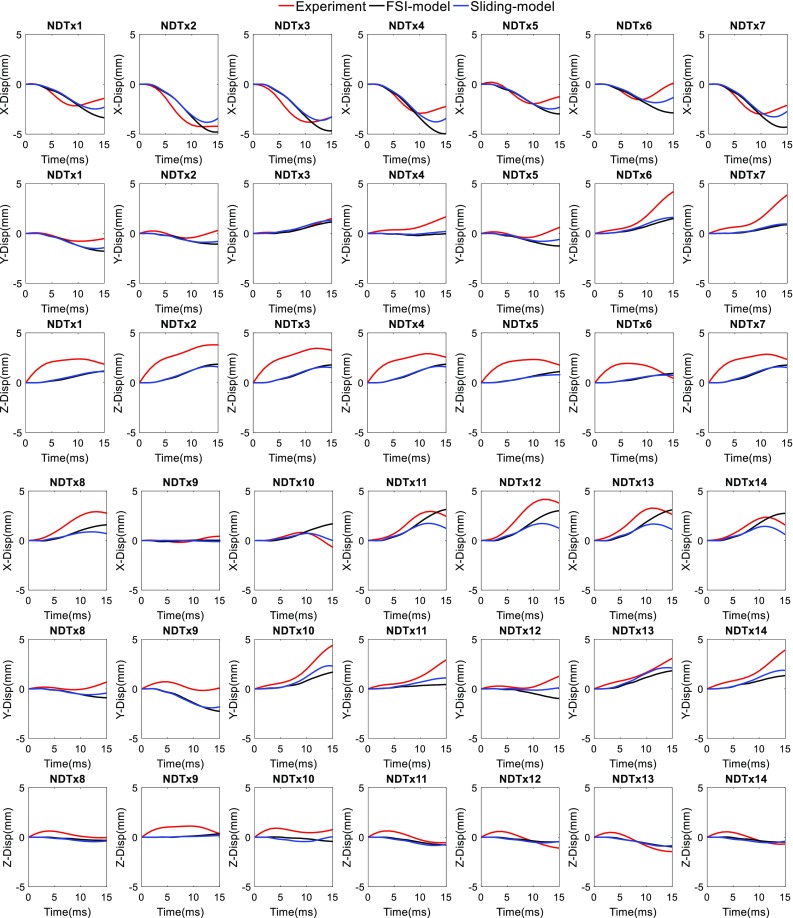
Comparison between experimental data and simulated brain–skull relative displacement for the experiment C380-T5

## Appendix A: Mesh sensitivity analysis

A convergence study is conducted on the dependence of the cortical relative motion, BV strain and brain strain responses on the element layer and aspect ratio of the ALE-represented CSF elements.

To determine the sensitivity of the head model response on CSF element layer, three models with varying element layers through the CSF thickness are created. The numbers of CSF fluid elements are 6243 for Model 1 with one layer of elements in the CSF thickness, 8015 for Model 2 with two layers of elements in the CSF thickness, and 9787 for Model 3 with three layers of elements in the CSF thickness (Fig. 9).

Of the three models, the element sizes of the CSF are the same as those of the cortical brain elements. To further investigate the sensitivity of the head model response on CSF aspect ratio, Model 4 is generated by splitting the CSF mesh in Model 2 by half in the in-plane directions. Thus, the aspect ratio of CSF elements in Model 4 is about half of that in Model 2.

The four models are subjected to two sinusoidal-shaped pulses with a peak value of 15 ${\text{krad}}/{\text{s}}^{2}$ for the rotational acceleration and 270 g for the translational acceleration in the posterior–anterior direction. Both pulses last for 5 ms (Fig. 10).

The results (Fig. 11) show that both the cortical relative motion and maximum BV strain are sensitive to the CSF discretization. When representing the CSF as one-layer elements, maximum cortical relative motion is found at the posterior frontal and precentral regions with a peak of 10.56 mm, consequently resulting in a BV strain peak of 0.340 at the same site. When increasing the element layer to two, maximum cortical relative motion is found at the precentral regions with a peak value of 9.56 mm. It is also notable that area with cortical relative motion over 8 mm extended backwards to the post-central regions at which the maximum BV strain (0.439) was measured. When further refining the CSF from two-layer elements to three-layer elements, no evident variations are found, either in the cortical relative motion distribution and peak (9.73 mm) or in maximum BV strain occurring site and its peak (0.429).

When dividing the CSF elements in the Model 2 by half in the in-plane direction, the cortical relative motion exhibits a similar pattern as that by Model 2. The peaks estimated by Model 4 are 9.82 mm for the cortical relative motion and 0.443 for the BV strain, respectively.

For the brain strain response, similar first principal strain distributions are noticed among the four models with an addition of Model 1 where high strain spreads at the cortical parietal region (Fig. 12).

Response similarity between Model 2 and Model 3 in terms of distribution and magnitude of cortical relative motion, BV strain and brain strain indicates that representing CSF as two-layer elements is adequate for the current study. Insignificant variation in the head responses between Model 2 and Model 4 implies that the aspect ratio in Model 2 is suitable to achieve converged brain response.

## Appendix B: Cut-off pressure sensitivity analysis

While modelling the CSF as fluid elements, the cut-off pressure is an important parameter limiting the amount of tensile pressure at the contrecoup site. The cut-off pressure for the CSF has been frequently assumed near-zero absolute pressure, supported by the claim that most fluids depend largely on external atmospheric pressure to hold them together due to the absence of cohesive strength. However, this claim needs further verification when it comes to the CSF, especially considering the existence of nucleation sites in the CSF may substantially raise the cavitation strength. Thus, a parametric study is conducted on the dependence of the intracranial pressure, brain strain and cortical relative motion, on the modelling choice of cut-off pressure for the CSF.

To achieve this, two models are developed with two levels of cut-off pressure level for the CSF: one with a value of − 22 MPa (cavitation strength of degassed water at 36 °C) and the other with a value of − 100 kPa (zero absolute pressure). Both models are subjected to the sinusoidal-shaped loading pulses in “Appendix A”.

Figure 13 shows the intracranial responses predicted by the models. When the cut-off pressure of the CSF is − 22 MPa, no cavitation occurs at 2.5 ms, i.e. the translational acceleration peaking time. At the same instant, brain strain over 0.2 is noticed at inferior region of the cerebral, probably due to the constrained effect by the basilar skull. The maximum cortical relative motion is observed at the posterior frontal and precentral region. When modifying the cut-off pressure of the CSF as − 100 kPa, the model predicts brain–skull decoupling at the countercoup site, with a maximum negative pressure close to − 100 kPa. Due to the lack of tensile resistance, the inertia force of the brain itself results in a much larger positive pressure at the coup site, compared to its counterpart simulation. Brain strain over 0.2 is noticed at the cerebral regions inferior to tentorium as well as posterior to the lateral ventricle. The maximum cortical relative motion is observed at the contrecoup site, extending backwards to the posterior frontal and precentral region.

Comparison of the contours of the intracranial pressure, brain strain as well as the brain–skull relative motion shows that the intracranial responses are sensitive to the choice of the cut-off pressure of the CSF.

## Appendix C: Validation results of brain–skull relative motion

See Figs. 14, 15, 16.

## References

[CR1] Aimedieu P, Grebe R (2004). Tensile strength of cranial pia mater: preliminary results. J Neurosurg.

[CR2] Al-Bsharat AS, Hardy WN, Yang KH, Khalil TB, Tashman S, King AI (1999) Brain/skull relative displacement magnitude due to blunt head impact: new experimental data and model. In: Proceedings of 43rd stapp car crash conference, Warrendale, PA. Society of Automotive Engineers, pp 321–332

[CR3] Aquelet N, Souli M, Olovsson L (2006). Euler-Lagrange coupling with damping effects: application to slamming problems. Comput Methods Appl Mech Eng.

[CR4] Batterbee D, Sims N, Becker W, Worden K, Rowson J (2011). Computational model of an infant brain subjected to periodic motion simplified modelling and Bayesian sensitivity analysis. Proc Inst Mech Eng [H].

[CR5] Benson DJ (1989). An efficient, accurate, simple ALE method for nonlinear finite element programs. Comput Methods Appl Mech Eng.

[CR6] Bloomfield I, Johnston I, Bilston L (1998). Effects of proteins, blood cells and glucose on the viscosity of cerebrospinal fluid. Pediatr Neurosurg.

[CR7] Bradshaw D, Morfey C (2001) Pressure and shear response in brain injury models. In: Proceedings of the 17th international technical conference on the enhanced safety of vehicles, Amsterdam, The Netherlands

[CR8] Bylski DI, Kriewall TJ, Akkas N, Melvin JW (1986). Mechanical behavior of fetal dura mater under large deformation biaxial tension. J Biomech.

[CR9] Chen Y, Ostoja-Starzewski M (2010). MRI-based finite element modeling of head trauma: spherically focusing shear waves. Acta Mech.

[CR10] Cheng J, Howard I, Rennison M (2010). Study of an infant brain subjected to periodic motion via a custom experimental apparatus design and finite element modelling. J Biomech.

[CR11] Claessens M, Sauren F, Wismans J (1997) Modeling of the human head under impact conditions: a parametric study. In: Proceedings of 41st Stapp Car Crash Conferences, Warrendala, PA. Society of Automotive Engineers, pp 315–328

[CR12] Cloots R, Van Dommelen J, Nyberg T, Kleiven S, Geers M (2011). Micromechanics of diffuse axonal injury: influence of axonal orientation and anisotropy. Biomech Model Mechanobiol.

[CR13] Cloots RJ, Van Dommelen J, Kleiven S, Geers M (2013). Multi-scale mechanics of traumatic brain injury: predicting axonal strains from head loads. Biomech Model Mechanobiol.

[CR14] Cui ZY, Famaey N, Depreitere B, Ivens J, Kleiven S, Vander Sloten J (2017). On the assessment of bridging vein rupture associated acute subdural hematoma through finite element analysis. Comput Methods Biomech Biomed Eng.

[CR15] Darvish KK, Crandall JR (2002) Influence of brain material properties and boundary conditions on brain response during dynamic loading. In: Proceedings international research council of biomechanics of injury, Munich, Germany. pp 339–350

[CR16] Depreitere B, Van Lierde C, Sloten JV, Van Audekercke R, Van Der Perre G, Plets C, Goffin J (2006). Mechanics of acute subdural hematomas resulting from bridging vein rupture. J Neurosurg.

[CR17] Dobratz B (1981) LLNL explosives handbook: properties of chemical explosives and explosives and explosive simulants. Lawrence Livermore National Lab., CA (USA)

[CR18] Fahlstedt M (2015) Numerical accident reconstructions: a biomechanical tool to understand and prevent head injuries. Dissertation, KTH Royal Institute of Technology

[CR19] Faul M, Xu L, Wald MM, Coronado VG (2010). Traumatic brain injury in the United States: emergency department visits, hospitalizations and deaths.

[CR20] Felippa C, DeRuntz J (1984). Finite element analysis of shock-induced hull cavitation. Comput Methods Appl Mech Eng.

[CR21] Fontenier B, Hault-Dubrulle A, Rahmoun J, Naceur H, Drazetic P, Fontaine C (2014). Experimental and numerical studies of fluid–structure interaction phenomena inside the head when subjected to a dynamical loading. Comput Methods Biomech Biomed Eng.

[CR22] Franceschini G, Bigoni D, Regitnig P, Holzapfel GA (2006). Brain tissue deforms similarly to filled elastomers and follows consolidation theory. J Mech Phys Solids.

[CR23] Gehre C, Gades H, Wernicke P (2009) Objective rating of signals using test and simulation responses. In: 21st International technical conference on the enhanced safety of vehicles, Stuttgart, Germany

[CR24] Ghajari M, Hellyer PJ, Sharp DJ (2017). Computational modelling of traumatic brain injury predicts the location of chronic traumatic encephalopathy pathology. Brain.

[CR25] Giordano C, Kleiven S (2016). Development of an unbiased validation protocol to assess the biofidelity of finite element head models used in prediction of traumatic brain injury. Stapp Car Crash Journal.

[CR26] Goriely A, Geers MG, Holzapfel GA, Jayamohan J, Jérusalem A, Sivaloganathan S, Squier W, van Dommelen JA, Waters S, Kuhl E (2015). Mechanics of the brain: perspectives, challenges, and opportunities. Biomech Model Mechanobiol.

[CR27] Gu L, Chafi MS, Ganpule S, Chandra N (2012). The influence of heterogeneous meninges on the brain mechanics under primary blast loading. Compos B Eng.

[CR28] Hallquist JO (2007). LS-DYNA keyword user’s manual.

[CR29] Hardy WN, Mason MJ, Foster CD, Shah CS, Kopacz JM, Yang KH, King AI, Bishop J, Bey M, Anderst W (2007). A study of the response of the human cadaver head to impact. Stapp Car Crash J.

[CR30] Herbert E, Balibar S, Caupin F (2006). Cavitation pressure in water. Phys Rev E.

[CR31] Ho J, von Holst H, Kleiven S (2009). Automatic generation and validation of patient-specific finite element head models suitable for crashworthiness analysis. Int J Crashworthiness.

[CR32] Ho J, Zhou Z, Li X, Kleiven S (2017). The peculiar properties of the falx and tentorium in brain injury biomechanics. J Biomech.

[CR33] Horgan T, Gilchrist MD (2003). The creation of three-dimensional finite element models for simulating head impact biomechanics. Int J Crashworthiness.

[CR34] Iwamoto M, Nakahira Y (2015). Development and validation of the Total HUman Model for Safety (THUMS) Version 5 containing multiple 1D muscles for estimating occupant motions with muscle activation during side impacts. Stapp Car Crash J.

[CR35] Jin X, Lee JB, Leung LY, Zhang L (2006). Biomechanical response of the bovine pia-arachnoid complex to tensile loading at varying strain-rates. Stapp Car Crash J.

[CR36] Jin X, Ma C, Zhang L, Yang KH, King AI, Dong G, Zhang J (2007). Biomechanical response of the bovine pia-arachnoid complex to normal traction loading at varying strain rates. Stapp Car Crash J.

[CR37] Jin X, Yang KH, King AI (2011). Mechanical properties of bovine pia–arachnoid complex in shear. J Biomech.

[CR38] Jin X, Mao H, Yang KH, King AI (2014). Constitutive modeling of pia–arachnoid complex. Ann Biomed Eng.

[CR39] Kimpara H, Iwamoto M (2012). Mild traumatic brain injury predictors based on angular accelerations during impacts. Ann Biomed Eng.

[CR40] Kleiven S (2003). Influence of impact direction on the human head in prediction of subdural hematoma. J Neurotrauma.

[CR41] Kleiven S (2006). Evaluation of head injury criteria using a finite element model validated against experiments on localized brain motion, intracerebral acceleration, and intracranial pressure. Int J Crashworthiness.

[CR42] Kleiven S (2007). Predictors for traumatic brain injuries evaluated through accident reconstructions. Stapp Car Crash J.

[CR43] Kleiven S, Hardy WN (2002). Correlation of an FE model of the human head with local brain motion: consequences for injury prediction. Stapp Car Crash J.

[CR44] Klug C, Sinz W, Brenn G, Feist F (2013) Experimental sphere-in-sphere testing for the validation of a numerical cerebrospinal fluid model. IRCOBI, Gothenburg, pp 483–496

[CR45] Larson B, Yang W, Ice G, Budai J, Tischler J (2002). Three-dimensional X-ray structural microscopy with submicrometre resolution. Nature.

[CR46] Lee M-C, Haut RC (1989). Insensitivity of tensile failure properties of human bridging veins to strain rate: implications in biomechanics of subdural hematoma. J Biomech.

[CR47] Li X, Sandler H, Kleiven S (2017). The importance of nonlinear tissue modelling in finite element simulations of infant head impacts. Biomech Model Mechanobiol.

[CR48] Lubock P, Goldsmith W (1980). Experimental cavitation studies in a model head-neck system. J Biomech.

[CR49] Luraghi G, Wu W, De Gaetano F, Matas JFR, Moggridge GD, Serrani M, Stasiak J, Costantino ML, Migliavacca F (2017). Evaluation of an aortic valve prosthesis: fluid-structure interaction or structural simulation?. J Biomech.

[CR50] Mao H, Yang KH (2011). Investigation of brain contusion mechanism and threshold by combining finite element analysis with in vivo histology data. Int J Numer Methods Biomed Eng.

[CR51] Mao H, Zhang L, Jiang B, Genthikatti VV, Jin X, Zhu F, Makwana R, Gill A, Jandir G, Singh A (2013). Development of a finite element human head model partially validated with thirty five experimental cases. J Biomech Eng.

[CR52] Miller LE, Urban JE, Stitzel JD (2016). Development and validation of an atlas-based finite element brain model. Biomech Model Mechanobiol.

[CR53] Nahum AM, Smith R, Ward CC (1977) Intracranial pressure dynamics during head impact. In: Proceedings of 21st Stapp car crash conference, Warrendale, PA. Society of Automotive Engineers, pp 337–366

[CR54] Nicolle S, Lounis M, Willinger R, Palierne JF (2005). Shear linear behavior of brain tissue over a large frequency range. Biorheology.

[CR55] Ning X, Zhu Q, Lanir Y, Margulies SS (2006). A transversely isotropic viscoelastic constitutive equation for brainstem undergoing finite deformation. J Biomech Eng.

[CR56] Panzer MB, Myers BS, Capehart BP, Bass CR (2012). Development of a finite element model for blast brain injury and the effects of CSF cavitation. Ann Biomed Eng.

[CR57] Peeters W, van den Brande R, Polinder S, Brazinova A, Steyerberg EW, Lingsma HF, Maas AI (2015). Epidemiology of traumatic brain injury in Europe. Acta Neurochir.

[CR58] Salzar RS, Treichler D, Wardlaw A, Weiss G, Goeller J (2017). Experimental investigation of cavitation as a possible damage mechanism in blast-induced traumatic brain injury in post-mortem human subject heads. J Neurotrauma.

[CR59] Scott GG, Coats B (2015). Microstructural characterization of the pia-arachnoid complex using optical coherence tomography. IEEE Trans Med Imaging.

[CR60] Scott GG, Margulies SS, Coats B (2016). Utilizing multiple scale models to improve predictions of extra-axial hemorrhage in the immature piglet. Biomech Model Mechanobiol.

[CR61] Souli M, Ouahsine A, Lewin L (2000). ALE formulation for fluid–structure interaction problems. Comput Methods Appl Mech Eng.

[CR62] Stitzel J, Duma S, Cormier J, Herring I (2002). A nonlinear finite element model of the eye with experimental validation for the prediction of globe rupture. Stapp Car Crash J.

[CR63] Van Leer B (1997). Towards the ultimate conservative difference scheme. J Comput Phys.

[CR64] Van Noort R, Black MM, Martin TRP, Meanley S (1981). A study of the uniaxial mechanical properties of human dura mater preserved in glycerol. Biomaterials.

[CR65] Willinger R, Baumgartner D (2003). Human head tolerance limits to specific injury mechanisms. Int J Crashworthiness.

[CR66] Zhang A, Suzuki K (2006). Numerical simulation of fluid–structure interaction of liquid cargo filled tank during ship collision using the ALE finite element method. Int J Crashworthiness.

[CR67] Zhang L, Bae J, Hardy W, Monson K, Manley G, Goldsmith W, Yang K, King A (2002). Computational study of the contribution of the vasculature on the dynamic response of the brain. Stapp Car Crash J.

[CR68] Zhao W, Ruan S, Ji S (2015). Brain pressure responses in translational head impact: a dimensional analysis and a further computational study. Biomech Model Mechanobiol.

[CR69] Zhou C, Khalil TB, King AI (1995) A new model comparing impact responses of the homogeneous and inhomogeneous human brain. In: Proceedings 39th stapp car crash conference, Warrendale, PA. Society of Automative Engineers, pp 121–137

[CR70] Zhou Z, Jiang B, Cao L, Zhu F, Mao H, Yang KH (2016). Numerical simulations of the 10-year-old head response in drop impacts and compression tests. Comput Methods Programs Biomed.

